# Alpha- and beta-band oscillations subserve different processes in reactive control of limb movements

**DOI:** 10.3389/fnbeh.2014.00383

**Published:** 2014-11-05

**Authors:** Pierpaolo Pani, Fabio Di Bello, Emiliano Brunamonti, Valeria D’Andrea, Odysseas Papazachariadis, Stefano Ferraina

**Affiliations:** ^1^Department Physiology and Pharmacology, Sapienza University of RomeRome, Italy; ^2^Center for Neuroscience and Cognitive Systems@UniTn, Istituto Italiano di TecnologiaRovereto (TN), Italy

**Keywords:** cognitive control, movement inhibition, stop task, monkey, reaching, beta-band, alpha-band, dorsal premotor cortex

## Abstract

The capacity to rapidly suppress a behavioral act in response to sudden instruction to stop is a key cognitive function. This function, called reactive control, is tested in experimental settings using the stop signal task, which requires subjects to generate a movement in response to a go signal or suppress it when a stop signal appears. The ability to inhibit this movement fluctuates over time: sometimes, subjects can stop their response, and at other times, they can not. To determine the neural basis of this fluctuation, we recorded local field potentials (LFPs) in the alpha (6–12 Hz) and beta (13–35 Hz) bands from the dorsal premotor cortex of two nonhuman primates that were performing the task. The ability to countermand a movement after a stop signal was predicted by the activity of both bands, each purportedly representing a distinct neural process. The beta band represents the level of movement preparation; higher beta power corresponds to a lower level of movement preparation, whereas the alpha band supports a proper phasic, reactive inhibitory response: movements are inhibited when alpha band power increases immediately after a stop signal. Our findings support the function of LFP bands in generating the signatures of various neural computations that are multiplexed in the brain.

## Introduction

Reactive control is the ability to rapidly suppress an imminent behavioral act in response to a sudden instruction to stop (Stuphorn and Emeric, 2012). It is a significant function in cognitive control that is impaired in many psychiatric diseases and genetic syndromes (Aron, 2011; Brunamonti et al., 2011; Takkar et al., 2011; Pani et al., 2013).

Reactive control is measured in experimental settings using the stop signal, or countermanding, task (Logan and Cowan, 1984). In most trials of this task, subjects must generate a movement in response to a go signal and inhibit the movement, as instructed by randomly presented stop signals. Typically, performance on the task fluctuates over time: sometimes, the subjects can stop their response and at other times fail, with the other task conditions being equal (Nelson et al., 2010).

A potential factor that favors this modulation in performance is the finding that stop signals are presented in brief periods that are characterized by various tonic levels of movement preparation. Changes in movement preparation are detected in oscillating reaction times (RTs) to the go signal during the task. Lower RTs are associated with greater movement preparation (or readiness to respond) and vice versa. At the neural level, a wide network that comprises frontal cortical and subcortical (especially the basal ganglia and cerebellum) structures regulates the competition between movement preparation and movement suppression, which constitute the two sides of movement control (Chambers et al., 2009; Stuphorn and Emeric, 2012; Brunamonti et al., 2014).

The computations of these structures can be examined by analyzing local field potentials (LFPs). Local field potentials reflect various subthreshold integrative processes, primarily synaptic inputs, that carry information about the state of the network and the local intracortical processing in the neural volume around the electrode tip (Mitzdorf, 1985; Logothetis, 2003; Kajikawa and Schroeder, 2011; Lindèn et al., 2011; Buzsáki et al., 2012). Local field potentials comprise several band-limited components—theta (θ: 4–7 Hz), alpha (α: 8–12 Hz), and beta (β: 13–35 Hz) (Ray et al., 2008)—that are associated with various functions and processing pathways. Thus, analyzing LFPs allows one to examine the presence of information channels that mediate the processing of neural information (Belitski et al., 2008; Montemurro et al., 2008; Kayser et al., 2009) that can not be detected by recording spiking activity (Logothetis, 2008).

Studies on the structures in the frontal-basal ganglia network that govern movement control support the diversity of information channels (Brittain et al., 2014). For example, at the onset of movement, motor cortices and basal ganglia structures experience a decrease in beta activity (Pfurtscheller et al., 2003; Zhang et al., 2008; Dejean et al., 2011; Jenkinson and Brown, 2011), whereas during holding-static periods, beta power rises (Baker et al., 1999, 2001; Williams and Baker, 2009). Alpha activity is related to the inhibition of the sensorimotor cortices (Pfurtscheller and Neuper, 1994; Suffczynski et al., 2001) and is considered a sign of top-down, cognitive inhibitory processing (Klimesch et al., 2007; Jensen and Mahazeri, 2010; Hwang et al., 2014). These band components thus represent distinct neural processes that coordinate motor control during a task or some phase of the task.

In this study, we examined the dynamic of the alpha and beta band components of LFPs while recording from the dorsal premotor cortex (PMd) of a monkey, an area that has significant function in the frontal basal ganglia network in motor control (Mirabella et al., 2011; Marcos et al., 2013). We found that the ability to countermand a movement after a stop signal is predicted by the activity of both bands: the alpha band supports a proper phasic, reactive inhibitory response, and the beta band regulates the level of movement preparation.

## Materials and methods

Two adult male rhesus monkeys (Macaca mulatta; monkey S ~7.5 Kg and monkey L ~8 Kg) were examined. All experimental procedures, animal care, housing, and surgical procedures conformed with European (Directive 86/609/ECC and 2010/63/UE) and Italian (D.L. 116/92 and D.L. 26/2014) laws on the use of nonhuman primates in scientific research and were approved (no. 58/2005-B) by the Italian Ministry of Health.

### Surgery, apparatus, and recording procedures

Under general anesthesia, a head-holding device, scleral eye coil (Robinson, 1963), and recording cylinder were implanted. The recording cylinder (18 mm in diameter) was stereotactically positioned on the left frontal lobe, over the right arm representation of the PMd (Paxinos et al., 2000). Recording positions were confirmed by structural MRI in monkey S and by visual inspection of anatomical landmarks after opening of the dura in monkey L. Details have been reported elsewhere (Mirabella et al., 2011; see also supplementary Figure 3).

The experiments were performed in a dim, sound-attenuated room. The monkeys were seated upright in a chair with the head fixed; the arm that was contralateral to the recorded hemisphere was free, and the other arm was restrained in a comfortable position.

A 21-inch PC monitor (CRT noninterlaced, refresh rate 85 Hz, 800 × 600 resolution, 32-bit color depth; monitor-eye distance 21 cm) that was equipped with a touchscreen (MicroTouch, sampling rate 200 Hz) was placed in front of the monkey to present stimuli and monitor touch positions. Visual stimuli consisted of red circles (2.43 cd/m^2^) with a diameter of 7.6° (2.8 cm) on a dark background of uniform luminance (<0.01 cd/m^2^). The stimuli were synchronized with the monitor refresh rate. A noncommercial software package, CORTEX ^1^, was used to regulate the stimuli and behavioral responses and collect neural (single unit activity 1 kHz) and eye movement (200 Hz) data.

Eye movements were monitored using a magnetic search coil technique (Remmel Labs, Ashland, MA, USA).

Neural activity was recorded extracellularly with a 7-channel multielectrode system (Thomas Recording, Giessen, Germany). The electrodes were quartz-insulated, platinum-tungsten fibers (80-µm diameter, 0.8 to 2.5-MΩ impedance) that were inserted transdurally, one at a time, with microdrives. After filtering and amplification steps (Thomas Recording, Giessen, Germany), a copy of the raw signal was sent to a dual-window spike discriminator (BAK Electronics, Mount Airy, MD, USA) for single-unit recording (online sorting). Single-unit results have been reported in a different format (Mirabella et al., 2011; Marcos et al., 2013). A second copy of the unfiltered raw signal was acquired with time stamps of the behavioral events for offline analysis (Tucker Davis Technologies, FL, USA; sampling rate 24.4 kHz). Modulation of high-frequency activity has been reported by Mattia et al. (2013).

### Behavioral task and analysis

**Figure 1:**
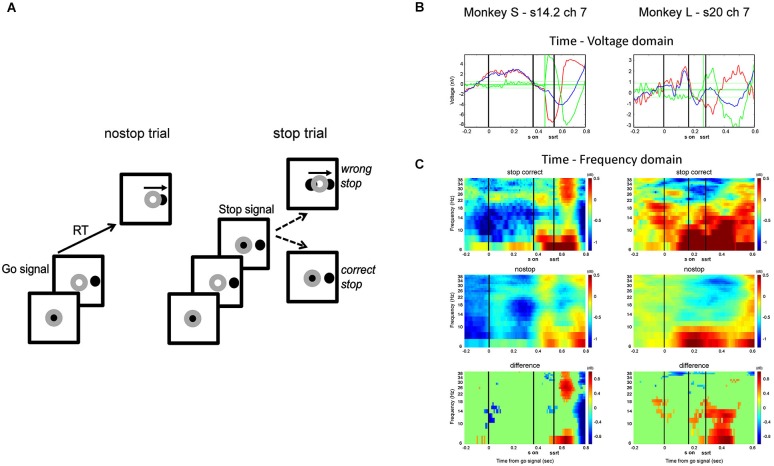
**(A)** Countermanding reaching task: each trial began with the hand (represented by the gray annulus) on the central black circle. After a random time, the central circle disappeared, and a target appeared in 1 of 2 opposite positions (Go signal; only one position is represented). In no-stop trials, the hand had to leave the resting position and touch the peripheral circle. In stop trials, after a variable delay (SSD) after the Go signal, the central circle reappeared (Stop signal), requiring the monkey to cancel the planned movement. **(B)** Two example channels that were selected for further analysis on the basis of their modulation. Red line represents the average activity of correct stop trials, the blue line is the average activity of latency-matched no-stop trials, and the green line is the difference between them. Horizontal green lines represent mean ± 2.5 SD of the difference between no-stop and correct stop trials in the 300 ms preceding the go signal (see text for further details). **(C)** Time-frequency plots showing the contribution of the beta and alpha bands in stop correct trials (top row) and no-stop latency-matched trials (middle row) and the difference between them (bottom row) for each monkey (S on, stop signal onset; SSRT is the estimated latency of the Stop Signal Reaction Time).

Each trial of the reaching countermanding task (Figure 1A) began with the appearance of a circle at the center of the screen that the monkeys had to touch and hold for varying times (500–800 ms). Then, the circle disappeared (Go signal), and simultaneously, a circle appeared at the periphery at one of two opposite positions. In the *no-stop trials*, the monkey had to reach for the target within an allotted time (RT-bound: 600 ms for monkey S and 750 ms for monkey M). In 33% of the trials (*stop trials*), the central circle reappeared unpredictably (Stop signal) after varying delays (stop signal delay, SSD), instructing the monkey to keep its hand in the resting position for at least 450 ms (650–850 ms for monkey S, 450–550 ms for monkey L).

A reward was delivered in the *no-stop* trials for initiating the reaching movement before the RT-bound was up and touching the peripheral circle; in *correct stop* trials, the reward was given for keeping the hand still in the initial position. No reward was delivered in *wrong stop trials*, when the monkey moved its hand despite a stop signal, even if the monkey change its mind and tried to return to the central circle.

To elicit an overall ability to inhibit of approximately 50%, we used either one of two techniques to adapt the SSDs to the monkeys’ behavior in the recording sessions: a fixed procedure, in which one of four delays was randomly presented in each stop trial, and a staircase procedure, in which the SSD increased (if the previous stop trial was correct) or decreased (if the previous stop trial was incorrect) by a fixed amount of time (step). In the fixed procedure, the SSDs were selected to effect the likelihood of inhibiting between 0.85 (for the shortest delay) and 0.15 (for the longest delay). In the staircase procedure, the step was 58.8 ms (five times the unit refresh rate).

We estimated the time it took each subject to cancel a movement—i.e., the stop signal reaction time (SSRT; Logan and Cowan, 1984)—by subtracting the *central* SSD (corresponding to a probability of inhibiting of ~50%) from the mean RT (mean method: Logan and Cowan, 1984; Hanes and Schall, 1995). For data on the fixed procedure, we obtained a function of inhibition, defined as the probability of inhibiting as a function of the SSDs. The inhibition function was then fitted with a Weibull cumulative function (see Mirabella et al., 2011 for further details on the same behavioral dataset) to generate the *central* SSD value, corresponding to 50% probability of inhibition. For data on the staircase procedure, we calculated the *central SSD*, representative of the overall runs, using the midrun estimation method (Wetherill and Levitt, 1965; Wetherill, 1966; Levitt, 1971). For the same dataset we calculated the SSRT with the integration method. For data obtained with the staircase procedure we considered only SSDs that were presented at least 14 times. Stop signal reaction time is calculated for each of the SSDs: RTs of no stop trials are rank ordered and the nth RT is found (nth = number of RTs × probability of having wrong stop trials at that SSD). The SSD is then subtracted from the nth RT, obtaining the SSRT (Logan and Cowan, 1984). The SSRTs obtained for each SSD are then averaged to compute a single SSRT estimate.

### Data analysis

We included every recording session that respected the following criteria in the database: overall inhibitory performance of 0.4–0.6 in at least one of the two movement directions and higher mean no-stop signal RT compared with the wrong stop RT. The latter comparison corresponds to a test of the fundamental assumption to calculate the SSRT (see Hanes et al., 1998; Mirabella et al., 2011 for further details). A lack of this assumption renders the data dispensable in calculating the SSRT (Logan and Cowan, 1984). For each dataset, we then considered the stop trials that corresponded to the SSD that was closer to the 50% probability to inhibit for each movement direction that respected the criteria above.

Once the data were chosen, based on the behavioral criteria, we narrowed them down on the basis of the neural signal. We included only channels that were artifact- and noise-free in the voltage domain. Moreover, we selected channels, based on their modulation of the LFP (voltage) task—i.e., signals that differed in voltage between the 300 ms preceding the Go signal (control epoch) and the RT epoch (task-related activity). Finally, we focused on channels that potentially governed the cancellation of fast movements (reactive control), because they are the only channels that can determine whether and when a movement is generated. To regulate the cancellation of fast (stimulus-driven) movements, a neural signal must have different task-related activities when a movement is generated vs. when it is canceled, and the change in activity must occur before the end of the estimated cancellation process (SSRT).

To determine whether our selected channels were involved in movement cancellation, we adopted similar criteria and methods as in previous studies (Hanes et al., 1998; Chen et al., 2010; Scangos and Stuphorn, 2010; Mirabella et al., 2011). For selected recordings, we compared LFP activity in the voltage domain of correct stop trials with that of latency-matched no-stop trials—i.e., no-stop trials with RTs longer than or equal to the sum of the SSD and SSRT. We computed the difference between signals of the two types of trials in the 300-ms epoch before the go signal. We set the threshold as the average of this difference plus 2.5 SD. The difference in average voltage between the two types of trials after the SSD and before the SSRT had to surpass the threshold for us to consider that the signal was potentially involved in movement cancellation.

This comparison controls for the level of movement preparation: the latency-matched no-stop trials are trials in which the movement would have been canceled if the stop signal had occurred, thus reflecting the same level of motor preparation in the correct stop trials. The probability of inhibition of ~50% in the stop trials permitted us to also select no-stop trial with a latency that matched those of wrong-stop trials.

Ultimately, the behavioral data set comprised 22 recording sessions (monkey *L* = 5; monkey *S* = 17) that respected the criteria above. For each of the recording sessions, we were able to record signals that passed the selection criteria—from up to six electrodes (channels), for a total of 63 channels (monkey *L* = 10; monkey *S* = 53).

Time-frequency analysis was computed using the multi-taper algorithm with the freeware toolbox “Chronux”^2^. For each trial, the mean value and linear trend were removed from the raw signal, and spectrograms were generated in a window of 300 ms with 10-ms steps, using a frequency bandwidth of 5 Hz and 2 Slepian tapers. We set the maximum frequency to 150 Hz and obtained a 131 × 97 time-frequency array, with frequency and steps of 1.5 Hz and 10 ms, respectively. Relative spectrograms were defined as the ratio (in dB) between power spectrum in each time-frequency bin and mean power spectrum across all trials of baseline activity between –300 ms and 0 ms relative to target onset. Each relative spectrogram corresponds to the average across all trials under the same conditions.

For each time-frequency bin, we computed the mean relative power spectrum across trials and analyzed the difference between mean spectra for the conditions of each pair of trials (e.g., successful stop trials vs. latency-matched no-stop trials). We examined whether the differences were significant by separate permutation tests for each bin. This test was performed by shuffling the power values across the two groups of trials, computing the difference in means between the reshuffled groups and repeating the shuffling process N times (*N* = 5000). For each comparison, we obtained a color-coded *p*-value map (*α* = 0.05) of differences by repeating the permutation test for all bins of the arrays. The green color of the resulting time-frequency maps indicates *p* > 0.05; other colors indicate sign and intensity of significant differences.

The overall significance was corrected by multiple comparison using the false-discovery rate (FDR) method (Benjamini and Yekutieli, 2001; Durka et al., 2004), with an *α*-value of 0.05. Briefly, this “step-up” method was performed by: (1) ordering the *p*-values in an ascending series—*p*_(1)_ ≤ *p*_(2)_ ≤ … ≤ *p*_(*k*)_; (2) finding the largest *k* for which *p*_(k) ≤_ *αk/m*; and (3) rejecting the null hypothesis for all bins with *p* ≤ *p*_(k)_.

We focused our analysis on two frequency bands: the upper part of the theta (6–7 Hz) and alpha bands (8–12 Hz)—hereafter called alpha—and the beta band (β: 13–35 Hz).

## Results

### Reactive control of movement

Monkey L reacted faster to the targets than monkey S in the no-stop trials (RTs in ms (mean ± std): 401.8 ± 62 and 555.6 ± 77, respectively) and wrong stop trials (379.3 ± 52 and 507 ± 50 respectively). There was a significant difference in RTs between no-stop trials and wrong stop trials in both monkeys (S: *t*-test (8327) = 15.02, *p* = 0.0001; *p* < 0.05); monkey (L: *t*-test (1518) = 3.5, *p* = 0.0005). We then considered a total of 84 SSDS across all the sessions and channels analyzed. For 73/84 (86%) of them the RTs in wrong stop trials were faster than those of no stop trials (non-parametric K-Smirnov test *p* < 0.05). These data confirmed the independence assumption of the race model (Logan and Cowan, 1984) and permitted us to reliably estimate the SSRT, yielding a behavioral measure of the reactive control of movement; for both monkeys, the probability of inhibiting when given a stop signal approached 50% (mean ± std, monkey L 0.49 ± 0.4 and monkey S 0.48 ± 0.9). The estimated speed overall in canceling the movement (SSRT) was ~140 ms (L: mean method, mean ± std132 ± 11; integration method, mean ± std 135 ± 19; S: mean method mean ± std 147 ± 17; integration method, mean ± std 149 ± 15). No difference was detected between the two estimates (K-Smirnov *p* > 0.1 in all comparisons). For analysis purpose we considered for each session the average of the two estimates.

We considered the modulation in voltage before the end of the SSRT to select the channels to analyze as follows. Figure 1B shows two representative channels (monkey L left, monkey S right) that were analyzed, Figure 1C shows the time-frequency power contribution of the alpha and beta bands to the observed signal in the voltage range (see Figure legend for further details).

### LFP signature of movement generation

Reaction times vary between trials. To determine the LFP correlates of such variation, we compared the RTs between the first one-third of the selected trials (ordered by RT duration) and third one-third, corresponding to the 33% fastest and 33% slowest RTs, respectively. Reaction times were longer in slowest trials (median ± std in ms: 632 ± 56 vs. 486 ± 45 in monkey S, and 432 ± 51 vs. 346 ± 50 in monkey L, *p* < 0.00001 in both cases, K-Smirnov test).

**Figure 2:**
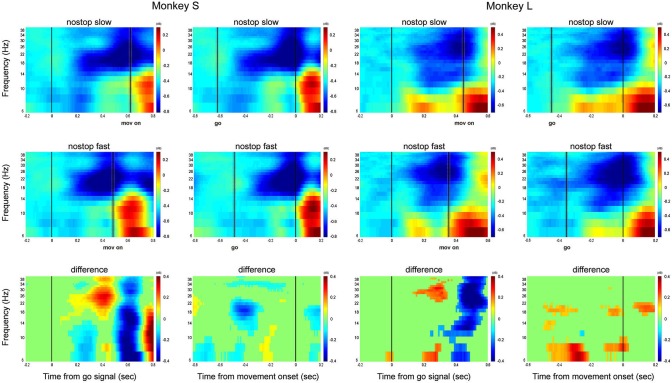
**Local field potential signature of movement generation**. Grand average time-frequency plot showing the LFP activity across all populations of recordings for each monkey. Left column for each monkey: alignment to the Go signal (go); right column: alignment to the movement onset (mov on). Top row: grand average of slowest responses. Middle row: grand average of fastest responses. Bottom row: difference between slowest and fastest responses. Dotted lines represent mean ± SD of RT in the left column and of the Go signal in the right column.

Figure 2 shows the time-frequency maps, relative to this analysis for each monkey separately). Approximately 100 ms after the go signal, beta activity declined in both monkeys (left columns for monkey L and S). Monkey L also showed an increase in alpha activity, locked to the go signal, whereas monkey S experienced a reduction in the same frequencies, followed by a slight increase.

Immediately prior to the start of the movement, the power across both frequencies declined. The difference between the two trial groups demonstrates that slower RTs are characterized in both monkeys by prolonged, higher beta activity following the go signal, and greater alpha activity.

When aligned to the onset of movement, the beta activity decreased immediately before the start of the movement and alpha activity rose in both monkeys (Figure 2, right columns for monkey L and S). The two conditions did not differ significantly in the 200 ms preceding the movement onset in both beta and alpha frequencies (bottom line).

**Figure 3:**
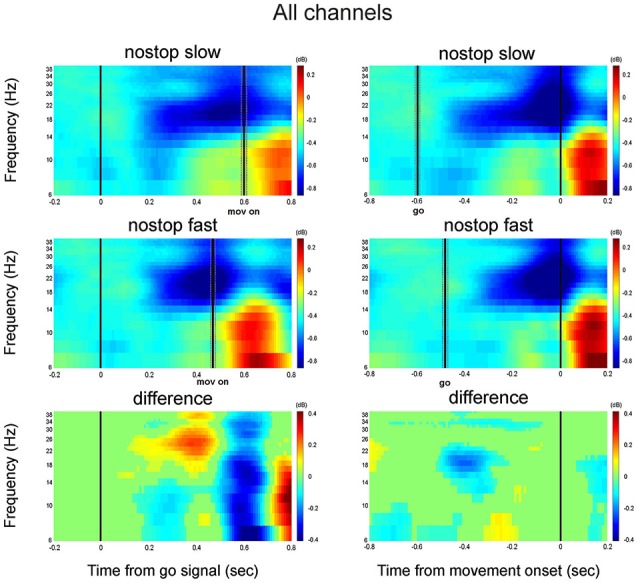
**Grand average time-frequency plot across all channels; Left column, alignment to the Go signal (go); right column: alignment to the movement onset (mov on)**. Top row: grand average of slowest responses. Middle row: grand average of fastest responses. Bottom row: difference between slowest and fastest responses. Other conventions as in Figure 2.

Thus, both monkeys were characterized by a more rapid decline in beta activity in the fastest trials, whereas alpha activity had opposite patterns between monkeys after the go signal and increased immediately after movement onset in both animals. Average across monkeys is illustrated in Figure 3. Trial by trial correlation analysis (Pearson correlation coefficients (Pcc) calculated separately for each channel), between each band mean power in the first RT epochs (from 50 to 250 ms after go signal) and the RT, showed a stronger positive correlation between RTs and beta activity compared to alpha (median Pcc 0.23 for beta and −0.21 for alpha, *p* < 0.0001, K-Smirnov test). The stronger relationship between beta activity and RTs was confirmed by the number of channels significantly modulated: 30/63 for beta and 13/63 for alpha (*Z*-test = 3.2, *p* = 0.00142, see Supplementary Figure 1).

The same analysis was performed on the last part of the epoch (from 200 before until RT). 16/63 channels showed a significant correlation between beta and RTs, while 13/62 between alpha and RT (*Z*-test = −0.64; *p* = 0.5; median Pcc 0.21 for alpha and 0.20 for beta, supplementary Figure 1, right column). No difference was observed between the two (K-Smirnov test, *p* = 0.99). Overall, in the first part of the trial, beta power showed a stronger correlation with RTs compared to alpha power, while just before the movement onset no difference was detected between the two frequencies.

### LFP signature of reactive cancellation

We examined LFP activity with regard to the reactive control of movement by selecting the correct stop trials with an SSD for which the probability of inhibiting was approximately 50% (minimum number of trials in wrong and correct stop = 8) and the corresponding latency-matched no-stop trials for each recording.

**Figure 4:**
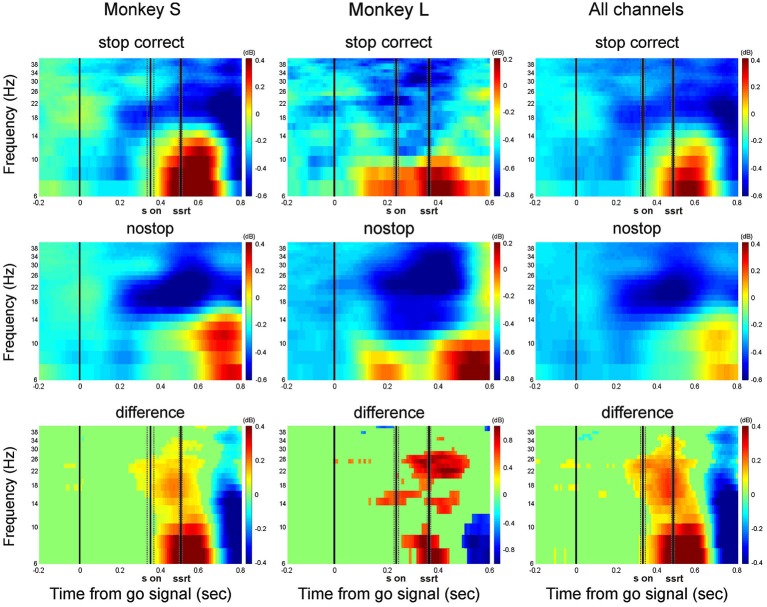
**Grand average of the time-frequency plots of correct stop trials (upper panels) and latency-matched no-stop trials (middle panels) and their difference (bottom panels)**. Data are presented aligned to the go signal separately for each monkey (Monkey S and Monkey L), and across all channels. Other conventions as in Figures 1, 2.

Figure 4 shows the contrasts between correct stop trials and latency-matched no-stop trials for monkeys L, S and for all channels recorded. The same comparison, referring to a single channel, has been shown in Figure 1C.

In comparing the two time-frequency maps, before the stop signal appeared, the activity was similar between correct stop trials (top row) and latency-matched no-stop trials (middle row). After the stop signal in stop correct trials, alpha band activity increased in both monkeys. Concurrently, the beta band did not decrease, as in latency-matched no-stop trials. The rise in alpha power and lack of decline in beta power occurred before the end of the SSRT, meeting the requirements for a signal to be considered as being involved in movement control. Thus in both cases, the reactive suppression of a movement was characterized by a phasic increase in alpha and a sustained beta activity before the end of the SSRT (see also supplementary Figure 2).

### LFP signature of successful vs. unsuccessful inhibition

To predict the effectiveness of reactive control, the described pattern should differ in wrong stop trials—i.e., trials in which the stop process is potentially driven but deficient in interrupting generation of the movement. Thus, we compared successful vs. unsuccessful inhibition.

As per the race model (Logan and Cowan, 1984), the failed inhibition in wrong stop trials is attributed to the level of motor readiness by the nervous system when the stop signal is presented: greater motor readiness effects a lower probability of suppressing the movement.

**Figure 5:**
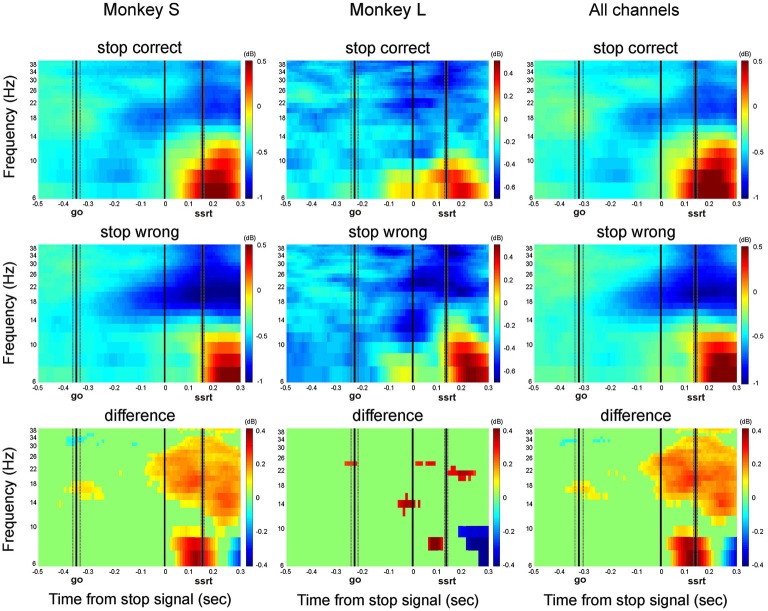
**Comparison between correct stop and wrong stop, aligned to stop signal presentation**. Conventions are as in Figure 4.

In comparing correct stop trials (Figure 5, top row) with wrong stop trials (middle row), the latter were characterized by low beta and low or delayed alpha activity when the stop signal appeared or immediately after. Beta and alpha activity was higher around the time when the stop signal was presented. This observation supports the hypothesis that these frequencies are linked to the result of the reactive control of movement.

In summary, the ability to interrupt a movement is characterized by two phenomena: a stimulus-driven stop process, reflected by an increase in alpha, and the level of motor readiness, represented by beta activity. In successful stop trials, beta activity remains high, and the stop signal drives the increase in alpha before the end of the SSRT, thus suppressing the reaching movement. In wrong stop trials, it appears that greater motor preparation is accompanied by ineffective instantiation of the stop process, represented by reduced and delayed alpha activity(see also supplementary Figure 2).

To evaluate the relationship between the two bands along the trial, we also performed a trial by trial correlation analysis (Pearson correlation coefficients calculated separately for each channel), between alpha and beta band power in the 150 ms preceding stop signal and from the stop signal to the end of the SSRT, in correct as well in wrong stop trials. We found that the two bands were overall weakly correlated. Before the stop signal presentation both in wrong and correct stop trials 17/63 channels showed a significant correlation between alpha and beta band. After stop signal presentation this number decreased to 13/63 in correct stop trials and to 11/63 in wrong stop trials. Thus the correlation between the two bands was observed only in a minority of channels (~20%), was not modulated by the phase of the task, and was not modulated by the type of trial (correct stop trials: *Z*-test = 0.8388, *p* = 0.40; wrong stop trials: *Z*-test = 1.29; *p* = 0.19; correct vs. wrong *Z*-test = 0.29, *p* = 0.76).

### Relationship between alpha and beta bands and the probability of success in stop trials

To further test the role of alpha and beta band activity in movement suppression, we asked whether alpha and beta band could vary depending on the probability of inhibition. We organized correct and wrong stop trials into three groups, depending on the difficulty or probability of success: easy trials, characterized by high probability of success (mean ± se: probability of success = 0.82 ± 12; SSDeasy = 262 ± 60); medium trials, were the stop trials with the SSD closer to the 0.5 probability of successful inhibition (probability of success 0.49 ± 0.11; SSDmedium = 354 ± 64); difficult trials, were the low probability of success trials, with SSDS longer than the previous one (mean ± se = 0.25 ± 0.14; SSDdifficult = 387 ± 8).

We then performed the analysis separately for alpha and beta band on 60/63 channels that provided enough trials (at least eight for each trial group), measuring the mean power recorded during the SSRT.

**Figure 6:**
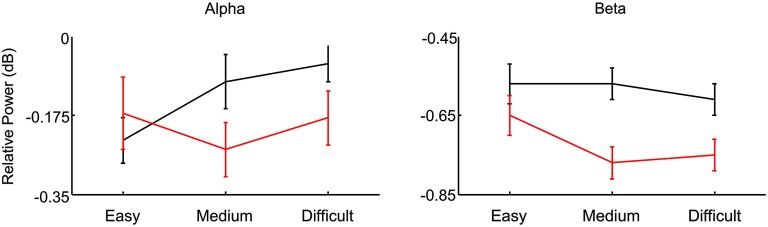
**Comparison between correct (black) and wrong (red) stop trials characterized by high (easy), medium or low (difficult) probability to inhibit the movement**. The comparison is done separately for alpha and beta band.

We found that correct stop trials showed a higher alpha activity compared to wrong stop trials for medium and difficult trials (power in dB (mean ± se): −0.1 ± 0.05 vs. −0.25 ± 0.06, *p* = 0.002, and −0.06 ± 0.04 −0.18 ± 0.06, *p* = 0.01 respectively, Figure 6), but no difference was observed for easy trials (power in dB (mean ± se): −3.23 ± 0.05 vs. −0.17 ± 0.07, *p* = 0.32). At the same time alpha activity of correct trials was lower in easy than in medium and difficult trials (*p* = 0.007 and *p* = 0.004 respectively), while no difference was observed between the last two (*p* = 0.31). In wrong stop trials no differences were detected between easy and medium or difficult trials (*p* = 0.2 and *p* = 0.8; repeated measures analysis with factors probability of success (easy, medium and difficult), and stop trial accuracy (correct, wrong): 2-way interaction decomposed: *F*_(2,118)_ = 8.07, *p* = 0.0005, Newman-Keuls *post hoc* test).

Beta activity showed a slightly different patterns: overall beta activity was higher in correct compared to wrong stop trials (main effect: *F*_(1,59)_ = 13,141, *p* = 0.0006; power in dB (mean ± se): −0.58 ± 0.06 vs. 0.72 ± 0.1, Figure 6); at the same time beta activity was higher in easy trials compared to medium and difficult trials, (power in dB (mean ± se): −0.61 ± 0.07 vs. −0.67 ± 0.06 and −0.68 ± 0.06 respectively (*F*_(2,118)_ = 3.7, *p* = 0.028), main effect of probability of success).

Overall these data shows that alpha band was higher in correct trials compared to wrong stop trials specifically in medium and difficult trials, and that was lower in easy correct trials. Beta activity was overall higher in correct stop trials than in wrong stop trials, and specifically higher in easy stop trials compared to medium and difficult trials.

### Comparison between wrong stop and latency matched no-stop trials

To detect subtle effect of the stop signal presentation in wrong stop trials, we compared wrong stop trials with latency matched no-stop trials, that is trials too fast to be inhibited had the stop signal been presented.

**Figure 7:**
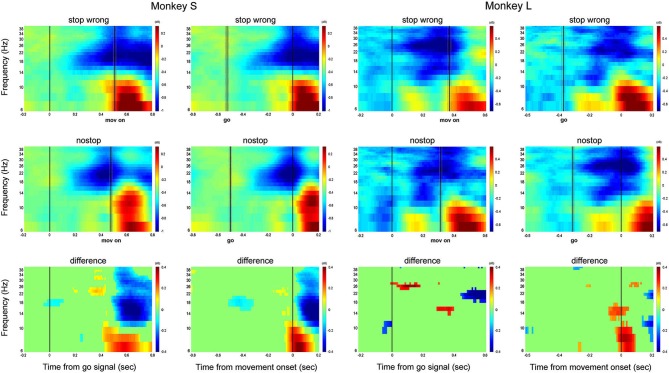
**Grand average time-frequency plot showing the LFP activity of wrong stop trials (upper panels) and latency-matched no-stop trials (middle panels) and their difference (bottom panels)**. Data are presented separately for each monkey. In left columns data are aligned to the go signal; in right colums are aligned to movement onset. Other conventions as in Figure 2.

**Figure 8:**
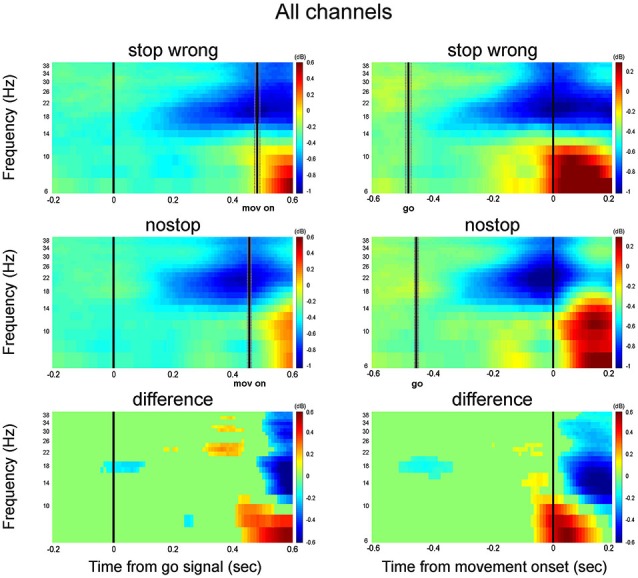
**Grand average time-frequency plot across all channels**. Other conventions as in Figure 6.

In comparing the two time-frequency maps after the go signal (Figure 7, left columns for monkey S and L; Figure 7 left column), the activity was similar between wrong stop trials (top row) and latency-matched no-stop trials (middle row). Toward movement onset a strong increase in alpha activity is observed for both trials type. This activity is clear when the maps are aligned to the movement onset (Figure 7, right columns for monkey S and L; Figure 8 right column). Importantly this activity rises first in wrong stop trials than in no-stop trials, and it is accompanied by a higher sustained beta activity.

### Tonic aspects of movement preparation affect the reactive control of movement

Changes in inhibitory performance are observed during the stop task. Further, correct stop trials occur at intervals of lower movement preparation in no-stop trials that immediately precede and follow them (Nelson et al., 2010), compared with no-stop trials before and after wrong stop trials. Thus, these tonic changes on the local temporal scale (the trials around the stop trial) can affect the conflict between movement generation and suppression.

To test this hypothesis, we analyzed the behavioral data. In both monkeys, the RTs in no-stop trials that preceded correct stop trials were slower compared with no-stop trials that preceded wrong stop trials (L: *p* = 0.002; S: *p* = 0.006). The same result was observed for no-stop trials that followed correct stop vs. wrong stop trials [L: 0.009; S: *p* = 0.002; factorial ANOVA with monkey (L and S), trial type (no-stop, stop correct, stop wrong), and time (no-stop trials immediately preceding or immediately following the trial type) as factors; 3-way interaction decomposed: *F*_(2,9698)_ = 5,6240, *p* = 0.004, Newman-Keuls *post hoc* test].

We then pooled the no-stop trials that surrounded the correct stop and compared them with no-stop trials that surrounded the wrong stop trials (mean ± sem: L: 419.9 ± 3.7 and 401.7 ± 3.7, respectively, *p* = 0.0004; S: 572.4 ± 2.2 vs. 552.95 ± 2.12, *p* = 0.00002, Newman-Keuls *post hoc* test).

**Figure 9:**
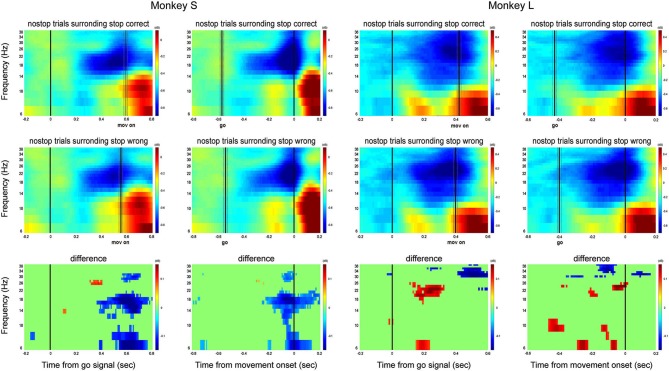
**Effects of tonic level of movement preparation: comparison between no-stop trials that surrounded correct stop trials and no-stop trials that surrounded wrong stop trials**. Conventions are as in Figure 2. Data are shown separately for each monkey.

**Figure 10:**
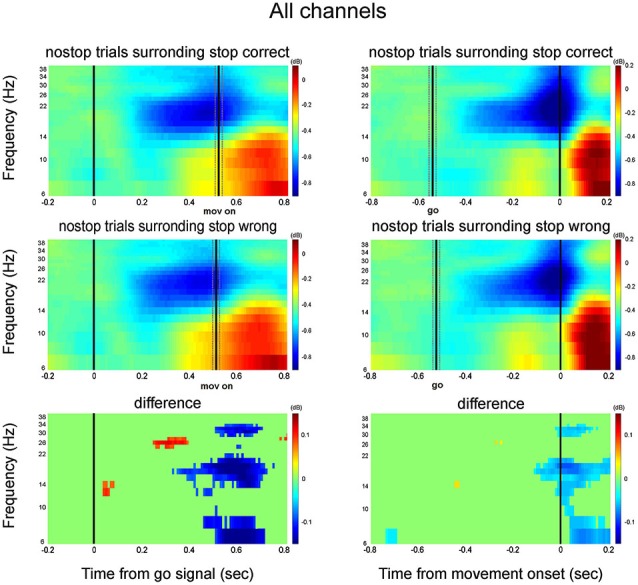
**Grand average time-frequency plot across all channels**. Other conventions as in Figure 9.

Thus, the behavioral data demonstrate that the level of movement preparation, represented by RTs, in the immediate context (i.e., preceding and following trials) contributes to determining behavioral performance. To determine the neural correlate of these fluctuations, we compared the no-stop trials that surrounded correct stop trials with those that surrounded wrong stop trials in each monkey. The decrease in beta activity characterized trials that surrounded wrong stop trials, assuming a pattern (Figures 9, 10) that was similar to that of the comparison between the slowest one-third vs. fastest one-third no-stop trials. These data support the function of beta activity in determining the readiness to respond, even on a local time scale.

## Discussion

We found that the ability to reactively control an imminent movement can be predicted by the level of beta- and alpha-band activities in the PMd. Specifically, the beta band appears to represent the activity of a sustained brake that affects a tonic level of motor preparation, whereas in this context, the alpha band reflects an inhibitory signal that characterizes the suppression of the movement, mostly with a phasic dynamic. Thus, the inhibition of a movement is related to these two distinct neural computations: one that represents a level of motor preparation, codified in the beta activity during the task, and the alpha activity that represents the phasic inhibitory signal that could suppress the movement.

The presence of alpha and beta bands modulations in the cortical and subcortical structures of the limb motor system has been established. Overall, our data and interpretations are consistent with recordings from motor cortices and with models on the functions of the various frequency components of the LFP (Gilbertson et al., 2005; Baker, 2007).

The inverse relationship between beta activity and the readiness to respond has been observed in humans and monkeys. One interpretation is that beta activity represents, at least in motor areas, the maintenance of a postural state and movement prevention (in our case, the hand maintained on the central target). During the holding period, strong beta activity has been noted in motor cortices (Baker et al., 1999, 2001; Williams and Baker, 2009). Also, an anticorrelation between beta activity and RTs has been reported in sensorimotor cortices and the supplementary motor area (SMA) in monkeys (Zhang et al., 2008; Chen et al., 2010). Gilbertson et al. (2005) found that movement acceleration was reduced when the cue that triggered the response was presented during times of elevated beta activity. Again, entraining cortical activity at 20 Hz in healthy subjects slows voluntary movement (Pogosyan et al., 2009). These observations and our data here support that beta activity participates to the stopping result by regulating the level of motor preparation.

The increase in beta activity that we observed in slower trials is attributed primarily to the input from the cortical and subcortical regions (Belitski et al., 2008) that affect the neural processes that promote movement generation through the cortical-basal ganglia-thalamocortical loop (Brown and Williams, 2005; Aron, 2011). These loops might also modulate alpha activity during the reactive control of movement. Increased alpha activity is linked to inhibition of the sensorimotor cortices (Pfurtscheller and Neuper, 1994; Suffczynski et al., 2001). A recent hypothesis suggests that this rhythm reflects top-down, cognitive inhibitory processing that supports the suppression of task-irrelevant processes and the competition between processes within the motor system (Klimesch et al., 2007; Jensen and Mahazeri, 2010). In our case, the increase in alpha activity was observed specifically after a stop signal and before the end of the SSRT.

The PMd participates in the frontal-basal ganglia-thalamofrontal network that governs movement control. Other frontal areas, such as the pre-SMA and SMA, have been implicated in forms of tonic (proactive) control, by modulating the level of responsiveness, and in reactive control, primarily by mediating the inhibition of responses. The signatures of proactive control in the pre-SMA and SMA are similar to the PMd—i.e., increased beta activity is associated with lower responsiveness. It is possible that the increase in beta is a general signal that affects many areas of the brain that participate in movement control, that probably originates in the basal ganglia (possibly striatum, Courtemanche et al., 2003; Zandbelt and Vink, 2010) and prefrontal cortex (Brown, 2007). In fact, robust synchronization has been observed between the beta rhythm in the subthalamic nucleus and motor structures (Kühn et al., 2009).

The involvement of the pre-SMA and SMA vs. PMd appears to differ in reactive control. Whereas single-cell recordings suggest that the PMd decides whether to generate a movement (Mirabella et al., 2011), the pre-SMA and SMA are more supportive of a disparate type of inhibitory signal (Chen et al., 2010; Scangos and Stuphorn, 2010). Specifically, neural activity in the pre-SMA might be a signal that is related to the motivation or tendency to inhibit. The reactive control can thus be triggered by lateral prefrontal regions (the right IFC in humans, Aron et al., 2014) that act through basal-ganglia structures (specifically, the subthalamic nucleus) on motor cortical structures, such as the PMd.

Thus, in the reactive control of movement, as evaluated by the countermanding task, a tonic level of motor readiness and a phasic inhibitory signal cohabit in the PMd.

We believe that the beta band constitutes the signature of a tonic brake that regulates the speed of movement preparation. Single-unit, as well as multiunit, activities in the PMd reflect the level of movement preparation and whether a movement will be performed (Mirabella et al., 2011; Mattia et al., 2013). Also, neural variability in this area is affected by the history of recent trials (Marcos et al., 2013). The beta and alpha bands can represent the correlates of two distinct synaptic computations that, ultimately, will act on the same neuronal populations to regulate behavior. The beta band seems to be related with the fine regulation of movement preparation, as confirmed also by the stronger correlation with RTs; while the alpha band with the sudden stop of the movement. Beta and alpha band correlates to each other only in about 20% of the analyzed channels, thus supporting the idea that in most cases they are independent.

A further support to the independence of the two computations comes from the modulation of their activity in easy, medium and difficult trials; alpha power is specifically higher in medium and difficult correct stop trials, while beta power is overall higher in correct stop trials, but specifically in the easy trials.

The possibility that neural computations subtending alpha and beta activity can act on the same neural populations is supported by the finding that neurons in the saccadic system are affected both by tonic (proactive) and reactive aspects of movement control (Pouget et al., 2011).

The stop signal task performance is normally analyzed in the frame of the race model. This model assumes that the go process, triggered by the go stimulus, and the stop process, triggered by the stop signal, race in parallel against each other. In stop trials the suppression of the movement will occur if the stop process wins the race. Attempts to find a neural instantiation of the go and stop process have identified go and stop units with movement and fixation neurons in the saccadic system (Boucher et al., 2007; Lo et al., 2009), and population of neurons in PMd (Mirabella et al., 2011). The modulations we observed could correlate with the activity of stop and go units, but not being identified with them. Specifically beta activity, that is anti-correlated with RTs, shows a temporal dynamic similar to that of a model of tonic inhibition that act on saccadic neurons and prevents from moving already at the presentation of the go signal (Lo et al., 2009). Alpha activity increases after stop signal presentation and before the end of SSRT in correct stop trials. This dynamic is similar to what observed in fixation neurons in saccadic centers and in some neurons in PMd (Hanes et al., 1998; Paré and Hanes, 2003; Mirabella et al., 2011), that probably instantiates the stopping process able to act (directly or indirectly) on movement neurons to suppress their activity. Alpha activity can thus be a correlate of the fast stopping process instantiated once the stop signal is presented.

These diverse neural rhythms likely reflect disparate functional pathways—one that acts when a sudden, fast change is required and the other that finely regulates the movement.

It is possible that these activities are sustained by partially overlapping structures, meaning that the same structures participate in different aspects of motor control through disparate brain rhythms. Similar machinery could allow different neural computations to be subserved by various frequencies, thus permitting parallel dispatches of information. This multiplexing might enhance the diversity of information that is sent to the neurons that mediate the final computation of the decision, allowing them to be regulated using more complex mechanisms.

Changes in LFP power detect episodic changes in the synchrony of certain frequency bands with regard to behavior. These oscillations might have small effects on behavior or none at all—a hypothesis that is related to the general idea that the neural computations that determine the behavior reside at the firing rate level (output of the neural population), whereas the oscillations can not affect them. Alternatively, it is generally accepted that modulations in LFP power reflect changes primarily at the synaptic level that contribute to alterations in the firing rate of neurons (Buzsáki et al., 2012).

Many studies support a causal role of oscillations on behavior. With regard to beta activity, for example, the stimulation of the motor cortex in healthy humans affects force production and the speed of responses (Pogosyan et al., 2009; Wach et al., 2013), and stimulation of the subthalamic nucleus in patients also decreases the speed of a response (Jahanshahi, 2013). There is a similar effect for alpha frequencies (Timmermann et al., 2004). Recently, a causal role of brain rhythms in regulating behavior has recently been demonstrated for higher (gamma) activities, as well. Engelhard et al. (2013) used a brain machine interface approach to train monkeys to move a cursor on a screen by modulating the gamma power recorded in motor cortex sites, thus showing that the volitional control of these frequencies generate sufficient information to perform a task.

We believe that the subsecond scale in which we observed the modulation constitutes sufficient time to affect behavior. Modulation in these frequencies can affect the activity of single neurons (Murthy and Fetz, 1992; Spinks et al., 2008), likely by modulating the firing activity or synchronized activity between neurons. At the level of the PMd, we have shown that weak inputs prime a chain reaction between neural modules (populations) from more excitable modules to others, promoting the development of a motor plan in approximately 120 ms (Mattia et al., 2013). Anyway this is hypothetical: a causal relationship between LFPs and behavior cannot be claimed in this study.

In our data, one monkey showed an increase in alpha activity after the appearance of a peripheral target, and alpha activity also rose after the reaching movement started. Can these observations do not support our interpretation of the alpha band as a correlate of a suppressive computation? We think this is not the case for some reasons.

The alpha modulation we observed and interpreted as a sign of suppression, occurs specifically during the SSRT and it is higher in stop correct compared to both no-stop and wrong stop trials. Also it occurs first in correct than in wrong stop trials, and it increases first in wrong stop trials compared to latency matched no-stop trials, purportedly representing an attempt of late suppression triggered by the stop signal presentation. The alpha modulation observed after the movement started can be interpreted in terms of a suppressive signal too. In fact at this moment of the task the monkey is reaching the peripheral target and will have to stop the hand on it to get the reward. Neurons in PMd are known to participate both in suppression and to the online updates of reaching movements (Battaglia-Mayer et al., 2014), and similar mechanism can be involved in inhibiting a being prepared as well as a being performed movement. We think that alpha activity we observed in monkey L is a response to the onset of the go signal instructing the reaching, a process that, is the same in both stop correct and no-stop trials. This phenomena, for frequency <10 Hz, has been already documented in both motor and premotor cortices (O’Leary and Hatsopoulos, 2006).

More generally, with regard to the issue of combining alpha activity as an inhibitory signal with these data, we believe that alterations in alpha activity can not be considered univocally a sign of motor or cognitive suppression, nor can other measures of the nervous system, such as firing rate, be considered specific cognitive functions. In fact the interpretation of the firing rate of a neuron will depend on the function that is subserved by the same neuron in a controlled context.

These frequency bands constitute a basic computation that can be used in many cognitive contexts. The function (in terms of behavior) must be established in specific experimental settings. It is possible, for example, that beta activity in this context reflects a basal-frontal system that regulates behavior finely, whereas alpha activity is a widespread braking mechanism that is implemented to halt a movement after the stop signal.

It is possible that these bands can be used to communicate between structures, thus forming the electrophysiological hardware, the content of which will depend on the structures that are active. It has been proposed that the cortex uses a multiplexing strategy to encode different types of information simultaneously on various time scales (Panzeri et al., 2010), thus increasing the capacity for information.

## Conflict of interest statement

The authors declare that the research was conducted in the absence of any commercial or financial relationships that could be construed as a potential conflict of interest.

## Footnotes

^1^http://dally.nimh.nih.gov

^2^http://chronux.org
