# Supplementary material for: Alpha- and beta-band oscillations subserve different processes in reactive control of limb movements
Source: Front Behav Neurosci. 2014 Nov 5;8:383. doi: 10.3389/fnbeh.2014.00383 (PMC4220745; doi:10.3389/fnbeh.2014.00383)
Supplement: Supplementary file 1 [file Data_Sheet_1.DOCX]

Alpha- and beta-band oscillations subserve different processes in reactive control of limb movements

Pani, P.^1^, Di Bello F.^1^, Brunamonti E.^1^, D’Andrea V.^1,2^, Papazachariadis, O. ^1^, and Ferraina S^1*^

^1^ Sapienza University of Rome, Dep. Physiology and Pharmacology

^2^ Center for Neuroscience and Cognitive Systems@UniTn, Istituto Italiano di Tecnologia, Via Bettini 31, 38068 Rovereto (TN)

^*^Correspondence: Prof. Stefano Ferraina, Sapienza University of Rome, Dep. Physiology and Pharmacology, Piazzale Aldo Moro 5, 00185 Rome, Italy

e-mail: stefano.ferraina@uniroma1.it

**Supplementary data**

We investigated whether if alpha and beta bands correlate with SSRT durations.

We calculated the Pearson correlation coefficient separately between each frequencies bands power in correct stop trials (from the stop signal presentation to the end of SSRT) and SSRTs in correct stop trials. We did not find significant correlations (Monkey S alpha-SSRT: Pearson Corr coeff=.01, p= 0.93; beta-SSRT Pearson Corr coeff=.05, p= 0.72; Monkey L:alpha-SSRT Pearson Corr coeff=.14, p= 0.68; beta-SSRT Pearson Corr coeff=.09, p= 0.77).

Although some study has shown a relationship between brain activity and SSRT length (cit. )

We did not detect a similar relationship; one possible explanation is that , in these over-trained animals, SSRT variability is too low to detect changes in alpha or beta power that correlates with the speed of inhibition.


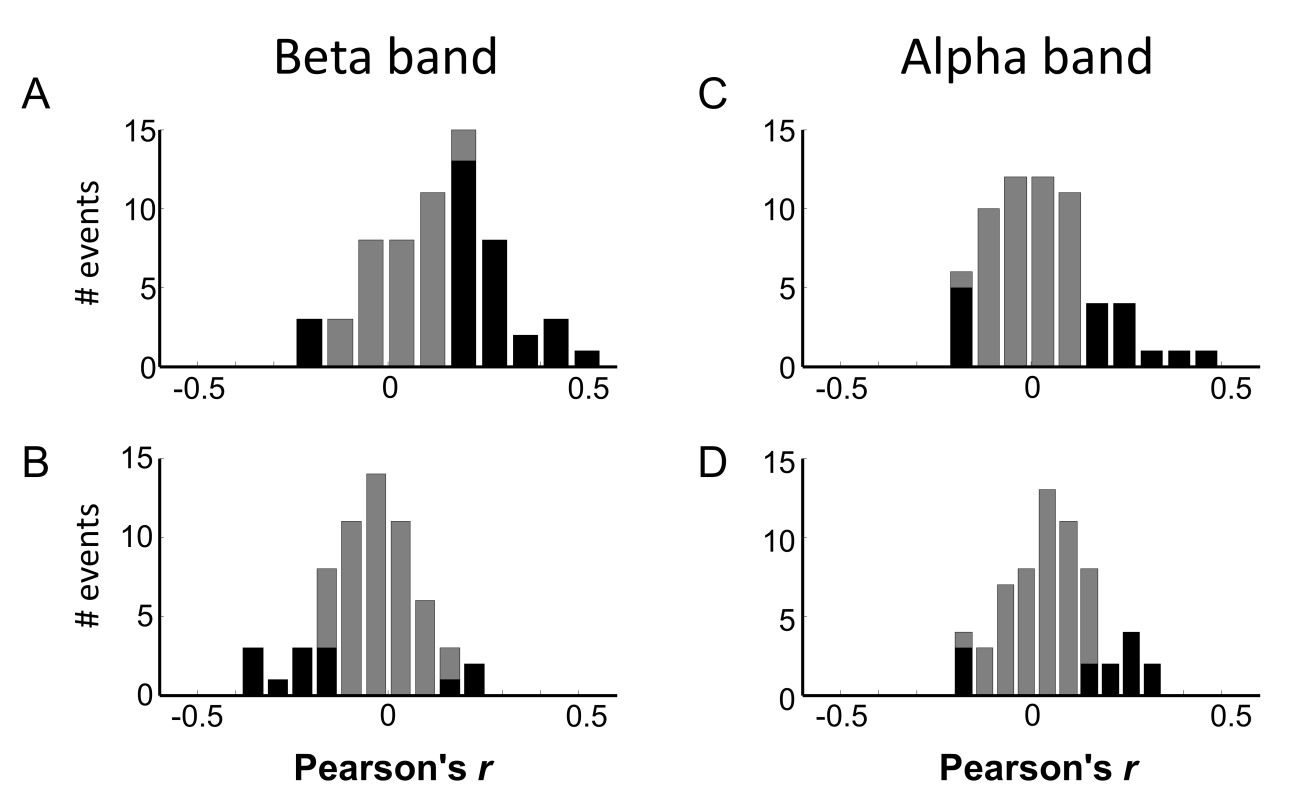


**Supplementary Figure 1**.

Correlation analysis between alpha and beta band power in no-stop trials and RTs. A: histogram of Pearson’s correlation coefficients between beta power in the epoch after go signal [+50 +300 ms] and RTs; B: histogram of Pearson’s correlation coefficients between beta band power in the epoch before RT[-200 0 ms].

Right column, top: Pearson’s correlation coefficients between alpha band in the epoch after go signal [+50 +250 ms] and RTs; bottom: Pearson’s correlation coefficients between alpha band in the epoch before RT. [-200 0 ms] . Black color identifies Pearson’s correlation coefficients significantly different from 0 (p<.01).


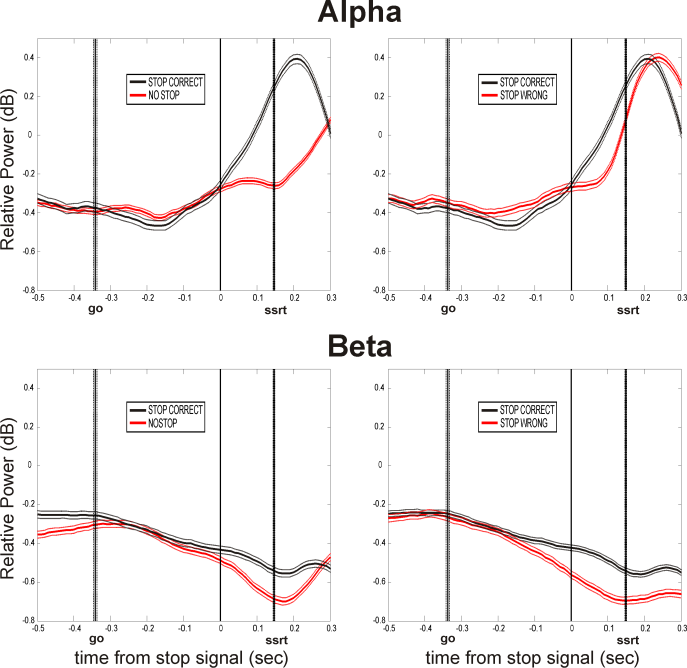


**Supplementary Figure 2**

Line graphs representations for the stop correct vs no-stop latency matched (left column) and stop correct vs stop wrong trials (right column) separately for alpha and beta band. Lines represent mean ±se ).


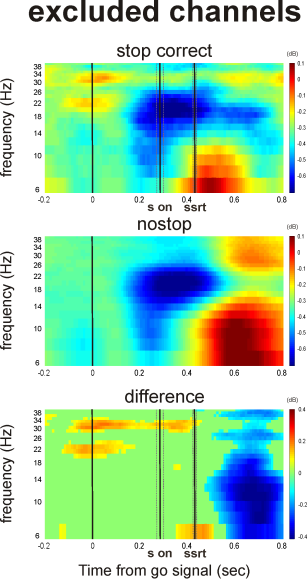


**Supplementary Figure 3**

Contrast between stop correct and no-stop trials of excluded channels. Grand average of the time-frequency plots of correct stop trials (upper panel) and latency- matched no-stop trials (middle panel) and their difference (bottom panel). Data are presented aligned to the go signal. The dataset to perform this analysis was composed by 41 channels obtained from 26 sessions. In these sessions behavior conformed to the requirement to calculate the SSRT (Monkey S 32 channels, Monkey L 19 channels) ,thus permitting the comparison between stop correct and latency matched no-stop trials. The difference map shows a higher level of beta activity already at the moment of go signal presentation in correct stop trials. After stop signal presentation beta activity is still high in correct stop trials and there is a slight increase around 6-7Hz before the end of SSRT. These changes are smaller if compared to the contrasts performed on channels participating in movement inhibition (see Fig. 4 in the main text).


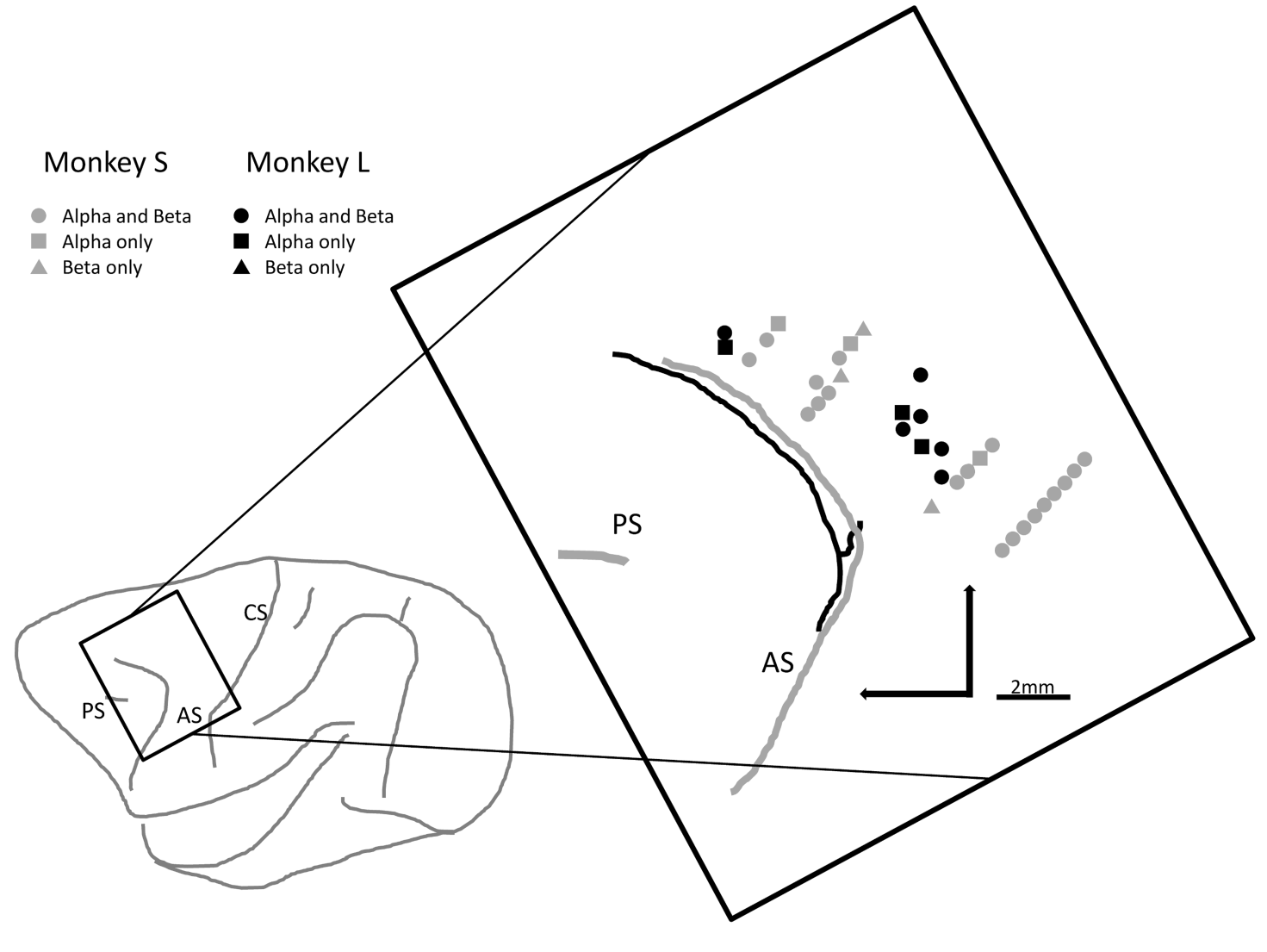


**Supplementary Figure 4**

Recording sites of the selected channels in the present study. The relative positions of the recording sites are indicated over a reconstructed model of rhesus monkey brain. Each monkey is codified by a different color. Different symbols are used to identify recording sites where comparison between correct stop and latency matched no-stop trials were significant for both alpha and beta power, for alpha power only or for beta power only


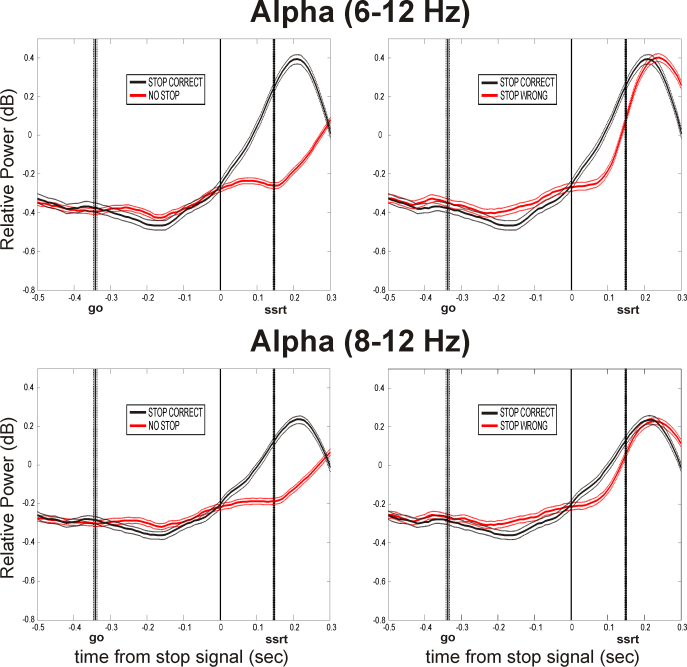


**Supplementary Figure 5**

Line graphs representations for the stop correct vs no-stop latency matched (left column) and stop correct vs stop wrong trials (right column) separately for alpha band as described in the manuscript (6-12Hz), and classic alpha band (8-12Hz). Lines represent mean ±se ).
